# Incident and Recurrent Depression among Adults Aged 50 Years and Older during the COVID-19 Pandemic: A Longitudinal Analysis of the Canadian Longitudinal Study on Aging

**DOI:** 10.3390/ijerph192215032

**Published:** 2022-11-15

**Authors:** Andie MacNeil, Sapriya Birk, Paul J. Villeneuve, Ying Jiang, Margaret de Groh, Esme Fuller-Thomson

**Affiliations:** 1 Factor-Inwentash Faculty of Social Work, University of Toronto, Toronto, ON M5S 1V4, Canada; 2 Institute for Life Course and Aging, University of Toronto, Toronto, ON M5S 1V4, Canada; 3 Department of Neuroscience, Carleton University, Ottawa, ON K1S 5B6, Canada; 4 CHAIM Research Centre, Carleton University, Ottawa, ON K1S 5B6, Canada; 5 Applied Research Division, Centre for Surveillance and Applied Research, Public Health Agency of Canada, Ottawa, ON K1A 0K9, Canada

**Keywords:** longitudinal study, depression, older adults, CLSA, COVID-19

## Abstract

The COVID-19 pandemic and accompanying public health measures have exacerbated many risk factors for depression in older adulthood. The objectives of the current study are: (1) to determine the risk of incident and recurrent depression during the COVID-19 pandemic among those with, or without, a history of depression; and (2) to identify factors that were predictive of depression in these two groups. The study population included 22,622 participants of the Canadian Longitudinal Study on Aging who provided data at baseline (2011–2015), follow-up (2015–2018), and twice during the pandemic (April–May 2020, September–December 2020). The Center for Epidemiologic Studies Depression Scale (CES-D-10) was used to classify individuals with depression. Logistic regression was used to estimate the odds of depression during COVID across a series of risk factors. Individuals with a history of depression had four times the risk of depression during the pandemic when compared to those without a history of depression, even after controlling for relevant covariates. Other factors associated with depression during the pandemic include being female, having fewer savings, and experiencing COVID-19 related stressors, such as health stressors, difficulties accessing resources, and family conflict. Clinicians working with older adults should consider interventions to support high-risk groups, such as those with recurrent depression.

## 1. Introduction

The COVID-19 pandemic and accompanying public health measures adopted to mitigate viral spread have profoundly impacted the mental health of individuals [1,2]. It has been suggested that the physical distancing and sustained shelter-in-place policies produced a “double pandemic” of COVID-19 and social isolation [3]. While older adults, aged 65 and above, generally report lower levels of depression than their younger counterparts [4,5], they have been particularly vulnerable to these policies. This is due to a number of factors, including increased risks of COVID-19 related morbidity and mortality [6], as well as more societal pressure relative to those of younger ages to strictly adhere to physical distancing guidelines [7,8]. Moreover, older adults are recognized to face greater challenges than younger adults with the use of technology that can enable them to stay better connected with loved ones and thereby lessening the harms from extended periods of isolation [9]. Emerging research has already indicated that older adults were more prone to social and loneliness during the COVID-19 pandemic when compared to younger age-groups [10,11].

Of particular concern are the deleterious effects that increases in loneliness may have on rates of depression among older adults [12,13]. Research conducted early in the pandemic among Canadian adults, 45 to 70 years of age, found that 71.0% of women and 67.4% of men reported feeling lonely in the preceding week [14]. There is extensive evidence that loneliness in later life is associated with both incident and recurrent depression [15,16,17], highlighting concern that the pandemic may contribute to incident depression among those with no history of the disorder, as well as recurrent episodes of depression among those with a lifetime history of depression. Older adults with a history of depression represent a particularly vulnerable subpopulation, as a previous history of depression is a leading risk factor for depression in later life [18,19,20,21].

There are several other sociodemographic risk factors for depression in older adulthood that have been impacted by the COVID-19 pandemic, including low socio-economic status at the individual level [22,23] and the neighbourhood level [24], multimorbidity [25], and being female [23,26]. Low-income individuals and communities have experienced disproportionate COVID-19 related morbidity and mortality, exacerbating psychological stress among those with low socioeconomic status [27,28]. Furthermore, older adults with multimorbidity have experienced significant increases in psychological distress early in the pandemic [29], likely due to the increased risk of severe-COVID-19 related outcomes, coupled with greater pressure to adhere to physical distancing measures to mitigate infection risk. In addition, older women have been found to have a higher risk of experiencing loneliness and social isolation during the COVID-19 pandemic compared to older men [14], suggesting that their vulnerability to depression may be heightened during the pandemic. Collectively, these factors emphasize the urgent need to examine the mental health of older adults during the COVID-19 pandemic, with particular consideration for these high-risk populations.

To date, much of the research on the mental health outcomes of the COVID-19 pandemic has relied on findings from cross-sectional studies. Cross-sectional studies are susceptible to participation bias and are limited in modelling pathways to depression. This makes it difficult to differentiate between those with incident and recurrent depression, and also limits examination on the effects of pre-pandemic factors on depression during the pandemic. To better understand the impact of the COVID-19 pandemic on depression among older adults and to address existing research gaps, herein we use a large Canadian longitudinal study of adults aged 50 years and older (hereafter older adults) to achieve the following objectives: (1) to determine the risk of incident and recurrent depression during the COVID-19 pandemic among those with, or without, a previous history of depression and (2) to identify factors that were predictive of developing depression during the pandemic in these same two groups of individuals. Based on the literature discussed above, we hypothesize that older adults with a history of depression are more vulnerable to experiencing depression during the COVID-19 pandemic relative to similarly aged adults with no history of depression. We also hypothesize that those who are female and are of lower socioeconomic status are more vulnerable to incident and recurrent depression during the COVID-19 pandemic.

## 2. Methods

### 2.1. Data Source

The Canadian Longitudinal Study on Aging (CLSA) is a national, longitudinal study designed to explore aging among Canadians. The CLSA consists of a random sample of 51,338 men and women recruited from predominantly urban centers of the 10 Canadian provinces, who were aged 45–85 years old at enrollment, were able to complete the questionnaires in English or French, were physically and cognitively able to participate independently, and provided informed consent for participation [30,31]. Residents of the Canadian territories and remote regions, individuals living on Federal or provincial First Nations reserves/settlements, full-time members of the Canadian Armed forces, and individuals who were institutionalized (including long-term care) were excluded from the CLSA.

The CLSA follows participants for 20 years, or until death, with data collection occurring every three years following a baseline questionnaire [31]. There are two cohorts within the CLSA: the comprehensive and tracking. Those in the comprehensive cohort lived within 25–50 km of one of the 11 data collection sites (DCS) located in seven provinces at baseline. The CLSA’s DCS are located in Victoria, Vancouver, Surrey, Calgary, Winnipeg, Hamilton, Ottawa, Montréal, Sherbrooke, Halifax, and St. John’s. They provided responses to the questionnaires through in-person home interviews and additional detailed information was collected during a DCS visit through physical examinations and biological specimens (blood and urine). Those in the tracking cohort lived within or near urban centers of the 10 provinces, and questionnaires were administered computer-assisted telephone interviews (CATI). Detailed descriptions on the CLSA design are published elsewhere [31].

To date, the CLSA has collected data from four waves of questionnaires: the baseline (2012–2014), follow-up 1 (2015–2018), COVID-19 baseline (hereafter Spring 2020), and COVID-19 exit (hereafter Autumn 2020). The baseline and follow-up 1 questionnaires collected data on demographics, physical and mental health, economic status, lifestyle and behaviour, and healthcare service utilization. The Spring 2020 and Autumn 2020 questionnaires were launched to investigate the effects of the pandemic on Canadian older adults and were completed through telephone and online surveys. These questionnaires asked additional questions regarding COVID-19 symptoms, the effect of COVID-19 on mental health, workplace changes due to COVID-19, and COVID-19 related behaviours (e.g., handwashing).

### 2.2. Sample

**Figure 1:**
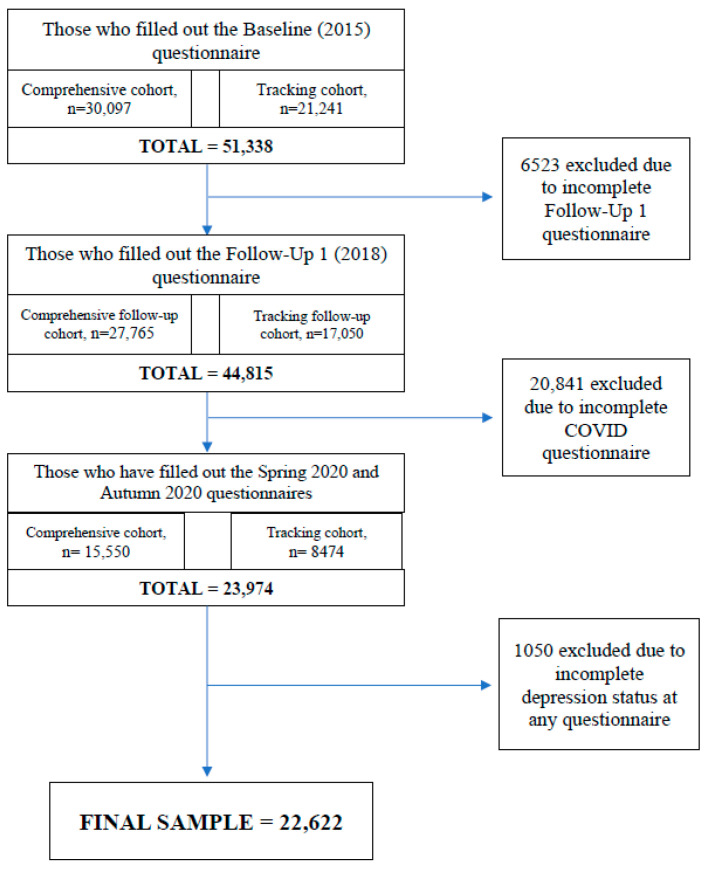
Study flowchart of CLSA participants used in this study.

This current paper focused on the outcome of depression during the COVID-19 pandemic among those with and without a history of depression. Figure 1 demonstrates the sampling strategy for this study. The 23,974 participants who responded to all four CLSA questionnaires were initially considered for inclusion. To identify those with a history of depression prior to COVID-19, participants needed to have screened positive for depression according to the Center for Epidemiological Studies-Depression scale (CES-D-10) [32] at baseline or follow-up 1 or responded affirmatively to the question “Has a doctor ever told you that you suffer from clinical depression?” at baseline or follow-up 1. There were 302 participants who were excluded due to missing responses to some of these questions. Lastly, 1050 participants with an incomplete CES-D-10 score at baseline, follow-up 1, Spring 2020, or Autumn 2020 were excluded. This resulted in a final sample size of 22,622.

### 2.3. Measures

#### 2.3.1. Outcome: Depression

Depression was measured using the short form of the Center for Epidemiological Studies—Depression (CES-D-10) scale [32,33] in all four questionnaires. The CES-D-10 is composed of ten questions asking participants to identify the frequency of feelings such as depression, loneliness, restlessness, happiness, and hopefulness. Responses to these questions are scored and summed to give a final score between 0 and 30. Depression was identified if the CES-D-10 score was equal to or greater than 10.

History of depression was identified using participants’ responses to the CES-D-10 and clinical depression diagnosis question. Participants were identified as having a history of depression if they screened positive for depression according to CES-D-10 at baseline or follow-up 1 and/or they responded affirmatively to the question “Has a doctor ever told you that you suffer from clinical depression?” at baseline or follow-up 1. Otherwise, they were considered as not having a history of depression. Those with a history of depression were further categorized into having recent or distant depression. Recent depression was defined as having a CES-D-10 score ≥ 10 at follow-up 1. Those with a history of depression that did not have a CES-D-10 score ≥ 10 at follow-up 1 were classified as having a distant history of depression.

#### 2.3.2. Covariates

Covariates included sociodemographic factors, health-related variables, social experiences and factors, and COVID-19 related stressors. Demographic factors included sex, age at COVID-19 baseline, marital status (single, married/common-law, separated/divorced/widowed), and immigrant status (born in Canada vs. not born in Canada). Socioeconomic status and wealth were measured by education, income, savings, and home ownership. The highest level of education achieved was categorized as less than secondary school, secondary school, some post-secondary education (trade school, non-university certificate, or university certificate below Bachelor’s), and post-secondary degree (Bachelor’s or degree above Bachelor’s). Total household income was defined as the income received by all household members, from all sources, in the past 12 months and was categorized as less than $20,000, $20,000–$49,999, $50,000–$99,999, $100,000-$149,999, and $150,000 or more. Participants were also asked about the total value of their savings and investments. This was categorized as less than $50,000, $50,000 to less than $100,000, $100,000 to less than $1 million, and $1 million or more. Home ownership was categorized as renting, own—with mortgage, own—mortgage paid off completely, own—mortgage status unspecified, and other. A subjective measure of wealth was also included. Participants were asked to indicate whether they felt that their income adequately satisfied their basic needs (adequate vs. inadequate).

Variables related to health included body mass index (BMI), chronic pain, and multimorbidity. BMI was categorized using the classification scheme used by the World Health Organization as underweight/normal weight (BMI < 25.0), overweight (BMI = 25.0–29.9), and obese (BMI ≥ 30.0). Chronic pain was identified by participants’ response to the question “Are you usually free of pain or discomfort” and was categorized into yes (usually have pain) and no (usually free of pain). Multimorbidity was categorized into those with no chronic conditions, those with one chronic condition, and those with two or more chronic conditions. These conditions included: included diabetes, heart disease (including congestive heart failure), peripheral vascular disease, memory problems, dementia or Alzheimer’s, multiple sclerosis, seizure disorders or epilepsy, migraine headaches, intestinal or stomach ulcers, and bowel disorders (including Crohn’s disease, ulcerative colitis, and irritable bowel syndrome).

Aspects of social support and social experiences were also briefly captured. Childhood adversities were measured and categorized as less than three and three or more of the following adverse childhood experiences: family history of mental/psychiatric illness, death or serious illness of parent/primary caregiver, and divorce or separation of parents before the age of 18. Experiences of any of the following before age 16 were also counted as a childhood adversity: physical, sexual, or verbal abuse, witness of verbal or physical abuse in the household, interaction with police or child protective services, and basic needs not being taken care of by parents, step-parents, or guardians. Participants were also asked if they had a household pet (yes vs. no). Lastly, a subjective measure of social inequality was measured by asking participants to rate where they think they stand in their community on a scale of 1 to 10, with 10 indicating the highest standing. Ratings were categorized as low (1–5), middle (6–7), and high (8–10).

At the Spring 2020 survey, participants were asked about their status as an essential worker (yes/no) and how many people they currently live with. A variable for living alone (yes/no) was created from responses to this question. Stressors related to COVID-19 were measured at the Autumn 2020 questionnaire. Participants were given twelve experiences/stressors and asked, “Which of the following stressors/experiences have you experienced during the COVID-19 pandemic?” The twelve stressors were categorized into five categories and participants were identified as having experienced that category of stressors if they responded yes to at least one experience. If they responded no to all experiences in a category, they were identified as not having that stressor. The five categories and corresponding stressors are listed: health stressors (“You were ill”, “People close to you were ill”, and/or “Death of a person close to you”), resource access (“Loss of income” and/or “Unable to access necessary supplies or food”), family conflict (Increased verbal or physical conflict” and/or “Breakdown in family/marital relationships”), other family issues (“Separation from family”, “Increased time caregiving”, and/or “Unable to care for people who require assistance due to health condition or limitation”), and healthcare (“Unable to access my usual healthcare” and/or “Unable to get my usual prescription medications and treatments”). Three measures of loneliness were also captured at Autumn 2020. Participants were asked how often they felt (1) that they lack companionship, (2) left out, and (3) isolated from others. They could respond “hardly ever”, “some of the time”, or “often”. Responses to these questions were coded into yes/no. If they responded “some of the time” or “often”, they were categorized as yes. If they responded “hardly ever”, they were categorized as no.

### 2.4. Statistical Analyses

All data management and statistical analyses were conducted using SPSS version 28.0.1.0 (142). Frequencies and percentages are reported categorically for all covariates. We tested for differences in the frequency distribution for these variables between those with and without a history of depression using the chi-square ($\chi^{2}$) statistic. Crude and adjusted odds ratios (ORs) with 95% confidence intervals (CIs) were calculated in logistic regression models, adjusted for age, sex, and marital status, and to determine which factors predict depression at COVID-19 among those with and without a history of depression, respectively.

Unconditional logistic regression was used to estimate the odds of having depression at Autumn 2020 for those with a history of depression, relative to those with no history. We repeated these analyses stratified by sex. In these models, depression at Autumn 2020 was a dichotomous outcome where the prevalence of depression was defined as having a CES-D 10 score of ten or higher. Those with scores less than ten were classified as not having depression at Autumn 2020. Separate models were fit to derive odds ratio for those with any history of depression, distant history of depression, and recent history of depression. Both crude and adjusted odds ratios were calculated. The adjusted model included the following categorical variables: age, sex, value of savings, marital status, chronic pain, experience of childhood adversity, loneliness (feeling left out), living alone during COVID-19, and COVD-19 related stressors. The selection of the adjustment factor was determined based on crude ORs associated with the outcome (depressed at Autumn 2020). However, to avoid over-adjusting, researchers used their discretion in choosing variables for inclusion in the adjusted model. For example, only one measure of wealth was included in the adjusted model (value of savings and investments). This covariate had the strongest crude association with depression at Autumn 2020 compared to the other wealth-related variables (i.e., household income, dwelling ownership, perception of income satisfying basic needs, etc.). Missing categories were not included in the model. Statistical significance was determined at a *p*-value < 0.05.

## 3. Results

### 3.1. Demographic Characteristics

The history of depression varied among socio-demographic characteristics (Table 1). Women were more likely to report a history of depression when compared to men (32.4% vs. 20.9%; *p* < 0.05). The history of depression was inversely related to household income, attained education, and other measures of wealth. In contrast, self-reported depression was lowed among those who were married or in common-law relationships. Those with other comorbid health conditions were also more likely to report a history of depression. Specifically, the history of depression among those with no chronic health conditions, one chronic health condition, or 2 or more chronic health conditions was 21.1%, 29.0% and 41.0%, respectively. Interestingly, those with a household pet were also slightly more likely to have a history of depression.

Similar descriptive analyses are presented for possible risk factors for depression during COVID-19 (Table 2). Nearly one quarter of all participants reported living alone, and they had a higher prevalence of depression when compared to those who did not (36.3% vs. 23.8%). There were no differences in the prevalence of depression among those who were essential workers compared to those who were not.

### 3.2. Depression during COVID-19 Pandemic

In this sample, 12.9% of participants with no history of depression developed depression during the COVID-19 pandemic. Comparatively, 45.2% of participants with a history of depression had depression at the time of their Autumn 2020 questionnaire. Over half of participants (58.2%) with a recent history of depression were depressed at their Autumn 2020 questionnaire.

Table 3 presents unadjusted and adjusted ORs with 95% CIs separately for those with and without a history of depression. Unadjusted ORs were used to help identify which factors to include in the logistic regression models. Adjusted ORs (aORs) were generated for each covariate to determine what factors may predict or contribute to the development of depression at the Autumn 2020 questionnaire. Our results indicate that women are more likely to have depression at Autumn 2020 for both those with a history of depression (aOR = 1.31 [1.18–1.46]) and without (aOR = 1.50 [1.36–1.65]). Among those without history of depression, the youngest (50–59) and oldest (90–96) age groups are at highest risk of having depression at Autumn 2020. Furthermore, a clear gradient is observed by value of savings and investments. Individuals with fewer savings are at greater risk of depression at Autumn 2020 compared to those with over $1 million in savings. The similar trend is shown for household income and highest education attained, although those results were not significantly different. Interestingly, owning a pet was associated with having depression at Autumn 2020.

As shown in Table 4, experiences of any COVID-19 stressors (health stressors, resource access, family conflict, other family issues, and healthcare) were associated with having depression at the Autumn 2020 questionnaire. The association between experience of family conflict and depression was the strongest for both participants without history of depression (aOR = 5.13 [4.45–5.92]) and with history of depression (aOR = 3.56 [3.05–4.15]). All three loneliness variables were also strongly associated with being depressed at Autumn 2020. Surprisingly, those who were essential workers during COVID-19 were at lower risk of being depressed compared to those who were not.

We found that a history of depression, as measured at follow-up 1, was strongly related to having depression at the Autumn 2020 questionnaire (Table 5). Specifically, those with a history of depression were nearly four times more likely more likely to have depression relative to those without a history of depression (aOR = 3.79, 95% CI: 3.48–4.12). The strength of this association was even stronger among those with a recent history of depression (aOR = 5.87, 95% CI: 5.27–6.55). Stratified analyses by sex revealed similar patterns in men and women.

## 4. Discussion

This longitudinal analysis examined depression among older Canadians with and without a history of depression during the COVID-19 pandemic. We found that older adults with a pre-pandemic history of depression were 5.6 times more likely to have depression during the COVID-19 pandemic when compared to those with no pre-pandemic history of depression. Controlling for relevant sociodemographics, health factors, and COVID-19 related adversities had only a modest attenuation on this relationship, as individuals with a history of depression were still 3.8 times more likely to have depression than those without a history of depression. This finding aligns with previous research that has found history of depression to be a leading risk factor for recurrence of depression in later life [18,19,20,21].

When examining differences in depression by recency of depression history, we found that individuals with a recent history of depression were 1.80 times more likely to have depression during the pandemic when compared to those with a distant history of depression. Older adults with a recent history of depression who experienced a recurrent episode of depression during the pandemic may represent a particularly vulnerable subset. Research on the recurrence of depression has found that experiencing a high number of depressive episodes in one’s lifetime and/or more severe depressive episodes are both associated with depression relapse and a shorter time to depression recurrence [34]. In addition, risk factors for recurring depressive symptoms in later life, such as loneliness [16] and stressful life events, including financial difficulties and relationship conflict [34] have been exacerbated by the pandemic [35,36].

Our analysis revealed several factors that were associated with both incident and recurrent depression among older adults during the pandemic. Unsurprisingly, individuals who experienced various dimensions of loneliness, such as feeling left out, feeling isolated, and lacking companionship had approximately 4 to 5 times higher risk of both incident and recurrent depression. This aligns with the substantial body of research that has found loneliness to be a major risk factor for both incident and recurrent depression among older adults [15,16,17]. These findings indicate that this pattern has persisted into the COVID-19 pandemic.

Older women were found to be more vulnerable to developing depression during the pandemic. Specifically, older women with no pre-pandemic history of depression were 1.54 times more likely to experience depression when compared to their male counterparts with no history of depression. Additionally, older women with a history of depression were 1.34 times more likely to experience recurrent depression than older men with a history of depression. Many studies have found that women are more likely than men to experience both incident and persistent depressive symptoms in later life [19,37]. Emerging research on mental health outcomes during COVID-19 indicates that this pattern has continued into the pandemic [38,39]. Lack of social support may partly explain the high vulnerability to depression among older women during the pandemic. Sonnenberg and colleagues [40] examined gender differences in the relationship between depression and social support in later life and found that low social support combined with a high need for social affiliation, such as the need to go to others for support during difficult times, was associated with depression among older women only. It is possible that the COVID-19 pandemic has led to a disparity between one’s need for social support and the accessibility of such support due to extended periods of lockdown and physical distancing. An additional study on gender differences in depression in older adulthood found that regular engagement in social outings acted as protective factor against depression for older women, but not for older men [41]. The ability to socialize in person was largely disrupted for extended periods of the pandemic, which may also influence the heightened risk of depression among older women.

There were several measures of socioeconomic status that were associated with depression among older adults during the pandemic, with some variation between those with and without a lifetime history of depression. For both incident and recurrent depression, these factors include having less than $100,000 in savings/investments in comparison to those with $1,000,000 or more in savings/investments, renting or owning a house with a mortgage compared to those who have paid off their mortgage, perceiving one’s income to be inadequate for satisfying their basic needs, and having difficulties accessing necessary resources during the pandemic. For recurrent depression only, completing less than secondary school was also associated with depression when compared to those counterparts with a post-secondary diploma. Research conducted prior to the pandemic has found socioeconomic disadvantage increases the odds of depression in later life, above and beyond demographic, lifestyle, social, and health factors [42,43]. Many of these socioeconomic risk factors may have been exacerbated by the economic precarity of the pandemic, particularly for individuals with less economic security. This may partially explain our finding that the risk of incident depression was lower among older adults who were essential workers when compared to those who were not essential workers. These individuals may have had greater economic stability during the early stages of the pandemic when compared to those who worked in industries that were disproportionately affected by periods of lockdown, such as retail and hospitality. Low assets and financial stressors during the pandemic have been found to be associated with higher odds of depression [44]. In contrast, having more assets, such as household savings, appear to afford protection against persistent depression symptoms during COVID [39]. These findings highlight the disproportionate mental health burden among individuals with low socioeconomic status during the pandemic.

### Limitations

There are some limitations that are important to consider when interpreting the findings of this analysis. First, depression was based upon the CES-D-10, a self-report measure. Although this measure has been found to have a 92% sensitivity with clinical interviews for identifying major depressive disorder [45], a psychiatrist assessment would have been preferable. Second, our sample did not include any individuals who had been residing in long-term care institutions during the baseline wave of data collection. When considering the extensive lockdowns in long-term care homes during the pandemic, it is likely that this population would have been particularly vulnerable to adverse mental health outcomes. As a result, the current study may underestimate the depressive symptoms in Canadian older adults by excluding this population. This also limits the generalizability of our findings to community-dwelling older adults. A further limitation is the lack of information on the exact onset of the depression. Although we know that respondents in the incident depression analysis were not depressed at or prior to the follow-up 1 wave, and that they were depressed in the autumn of 2020, we do not have information on the exact timing of the onset. It is possible that some individuals in this group developed depression after the follow-up 1 wave but before the COVID-19 pandemic. Thus, it is more helpful to think of this incidence as “period incidence.” Finally, the current study was only able to examine depression relatively early in the COVID-19 pandemic. While this period likely represents one of the most challenging times of the pandemic, given the severity of public health restrictions in many regions at this time, it is likely that some individuals would have experienced incident or recurrent depression later in the pandemic. Future longitudinal research should examine mental health patterns throughout various stages of the pandemic.

## 5. Conclusions

Despite these limitations, this longitudinal analysis provides valuable insights about depression among Canadian older adults during the COVID-19 pandemic. This study illuminates some of the key differences in depression risk between those with and without a history of depression, and also emphasizes the role that pre-pandemic factors play in the likelihood of experiencing depression during the pandemic. Vulnerable subpopulations among older adults, such as those with previous depression, women, and those with low socioeconomic status, are bearing a substantial mental health burden during the COVID-19 pandemic. By identifying subgroups of older adults with a heightened vulnerability to depression during the pandemic, the current study highlights the importance of targeted interventions to support these individuals. The under-treatment of depression, particularly persistent depression in older adults, is an ongoing public health issue [46,47]. It is essential to address the potential longstanding effects of the COVID-19 pandemic on the mental health of older adults.

## Figures and Tables

**Table 1:** History of depression across socio-demographic characteristics.

Characteristics		Total (*n* = 22,622)		Prevalence of History of Depression ^a^	No History of Depression (*n* = 16,525)		History of Depression (*n* = 6097)		*p*-Value
		n	%	%	n	%	n	%	
Sex	Male	10,583	46.8	20.9	8382	50.7	2201	36.1	
	Female	12,039	53.2	32.4	8143	49.3	3896	63.9	<0.001
									
Age-group	50–59	4183	18.5	28.1	3006	18.2	1177	19.3	
	60–69	8167	36.1	28.8	5817	35.2	2350	38.5	
	70–79	6629	29.3	24.8	4985	30.2	1644	27.0	
	80–89	3387	15.0	24.9	2542	15.4	845	13.9	
	90–96	256	1.1	31.6	175	1.1	81	1.3	<0.001
									
Household income	<20,000	777	3.4	49.7	391	2.4	386	6.3	
	20,000–<50,000	4514	20.0	35.0	2936	17.8	1578	25.9	
	50,000–<100,000	8143	36.0	26.2	6012	36.4	2131	35.0	
	100,000–<150,000	4397	19.4	21.7	3442	20.8	955	15.7	
	≥150,000	3597	15.9	18.6	2927	17.7	670	11.0	
	Missing	1194	5.3	31.6	817	4.9	377	6.2	<0.001
									
Highest attained education	Less than secondary school	173	0.8	37.0	109	0.7	64	1.0	
	Secondary school	1453	6.4	28.6	1038	6.3	415	6.8	
	Some post-secondary education	7142	31.6	29.1	5064	30.6	2078	34.1	
	Post-secondary degree	10,552	46.6	24.0	8016	48.5	2536	41.6	
	Missing	3302	14.6	30.4	2298	13.9	1004	16.5	<0.001
									
Value of savings and investments	<50,000	4076	18.0	37.4	2552	15.4	1524	25.0	
	50,000–<100,000	3238	14.3	30.1	2261	13.7	977	16.0	
	100,000–<1 million	11,285	49.9	23.9	8589	52.0	2696	44.2	
	≥1 million	2424	10.7	17.2	2008	12.2	416	6.8	
	Missing	1599	7.1	30.3	1115	6.7	484	7.9	<0.001
									
Dwelling ownership	Renting	2751	12.2	40.9	1627	9.8	1124	18.4	
	Own–with mortgage	5994	26.5	29.2	4242	25.7	1752	28.7	
	Own–mortgage paid off completely	13,505	59.7	22.9	10,408	63.0	3097	50.8	
	Own-mortgage unspecified	63	0.3	20.6	50	0.3	13	0.2	
	Other	233	1.0	33.9	154	0.9	79	1.3	<0.001
									
Immigrant status	Born in Canada	19,089	84.4	27.3	13,869	83.9	5220	85.6	
	Not born in Canada	3531	15.6	24.8	2654	16.1	877	14.4	0.002
									
Marital Status	Single	1871	8.3	38.2	1156	7.0	715	11.7	
	Married/Common-law	15,969	70.6	22.8	12,329	74.6	3640	59.7	
	Separated/Divorced/Widowed	4768	21.1	36.5	3030	18.3	1738	28.5	<0.001
									
Perceived community standing	1–5	6905	30.5	32.5	4664	28.2	2241	36.8	
	6–7	7696	35.2	26.3	5870	35.5	2099	34.3	
	8–10	6985	30.9	21.7	5469	33.1	1516	24.9	<0.001
									
Perception of income satisfying basic needs	Adequate	21,184	93.6	25.0	15,881	96.1	5303	87.0	
	Inadequate	1340	5.9	56.2	587	3.6	753	12.4	<0.001
									
BMI	Underweight/normal weight	7030	31.1	24.1	5334	32.3	1696	27.8	
	Overweight	9164	40.5	24.8	6893	41.7	2271	37.2	
	Obese	6248	27.6	33.0	4184	25.3	2064	33.9	<0.001
									
Chronic Pain	Yes	7638	33.8	38.3	4710	28.5	2928	48.0	
	No	14,953	66.1	21.1	11,795	71.4	3158	51.8	<0.001
									
Multimorbidity	No chronic conditions	11,570	51.1	21.1	9131	55.3	2439	40.0	
	1 chronic condition	7057	31.2	29.0	5010	30.3	2047	33.6	
	2 or more chronic conditions	3766	16.6	41.0	2221	13.4	1545	25.3	<0.001
									
Childhood adversities	Less than three	11,629	51.4	20.9	9201	55.7	2428	39.8	
	Three or more	9709	42.9	33.1	6498	39.3	3211	52.7	
	Missing	1284	5.7	35.7	826	5.0	458	7.5	<0.001
									
Household pet	Yes	8868	39.2	29.7	6233	37.7	2635	43.2	
	No	13,731	60.7	25.1	10,278	62.2	3453	56.6	<0.001

^a^ History of depression was identified at the time of Follow-Up 1 questionnaire.

**Table 2:** History of depression across COVID-related factors.

COVID-Related Factor ^a^		Total *(n* = 22,622)		Prevalence of History of Depression	No History of Depression (*n* = 16,525)		History of Depression (*n* = 6097)		*p*-Value
		*n*	%	%	*n*	%	*n*	%	
**Living alone**	Yes	5527	24.8	36.3	3520	21.3	2007	32.9	
	No	16,716	75.2	23.8	12,732	77.0	3984	65.3	<0.001
									
Essential worker	Yes	2396	10.6	26.0	1773	10.7	623	10.2	
	No	3279	14.5	26.4	2412	14.6	866	14.2	
	Missing	16,947	74.9	27.2	12,339	74.7	4608	75.6	0.354
									
COVID health stressors	Yes	7295	32.4	31.9	4967	30.1	2328	38.1	
	No	15,222	67.6	24.5	11,490	69.5	3732	61.2	<0.001
									
COVID resource access	Yes	3660	16.3	32.8	2458	14.9	1202	19.7	
	No	18,857	83.7	25.8	13,999	84.7	4858	79.7	<0.001
									
COVID family conflict	Yes	1992	8.8	45.0	1095	6.6	897	14.7	
	No	20,525	91.2	25.2	15,362	93.0	5163	84.7	<0.001
									
COVID other family issues	Yes	12,811	56.9	28.7	9133	55.3	3678	60.4	
	No	9706	43.1	24.5	7324	44.3	2382	39.1	<0.001
									
COVID health care	Yes	5514	24.5	32.6	3718	22.5	1796	29.5	
	No	17,003	75.5	25.1	12,739	77.1	4264	69.9	<0.001
									
Lack companionship	Yes	8377	37.1	39.7	5049	30.6	3328	54.5	
	No	14,191	62.9	19.4	11,441	69.2	2750	45.1	<0.001
									
Feeling left out	Yes	6345	28.2	42.2	3668	22.2	2677	43.9	
	No	16,119	71.8	20.8	12,764	77.2	3355	55.0	<0.001
									
Feeling isolated	Yes	9584	42.5	40.0	6041	36.5	3543	58.1	
	No	12,960	57.5	19.4	10,440	63.2	2520	41.3	<0.001

^a^ Living alone and essential worker status were captured at COVID-19 baseline. COVID-19 related stressors and loneliness variables were captured in Autumn 2020.

**Table 3:** Predictors of depression at the Autumn 2020 questionnaire among those with no history of depression and those with any history of depression.

Characteristics		No History of Depression (*n* = 16,525)		History of Depression (*n* = 6097)	
		Crude OR	Adjusted OR ^a^	Crude OR	Adjusted OR ^a^
Sex	Male (ref.)	1.00	1.00	1.00	1.00
	Female	**1.54 (1.40–1.69)**	**1.50 (1.36–1.65)**	**1.34 (1.21–1.49)**	**1.31 (1.18–1.46)**
					
Age-group	50–59	**1.38 (1.21–1.57)**	**1.37 (1.20–1.57)**	**1.34 (1.15–1.55)**	**1.35 (1.16–1.58)**
	60–69	1.07 (0.96–1.21)	1.07 (0.95–1.20)	1.10 (0.97–1.25)	1.12 (0.98–1.27)
	70–79 (ref.)	1.00	1.00	1.00	1.00
	80–89	1.14 (0.99–1.32)	1.13 (0.97–1.31)	1.12 (0.95–1.33)	1.09 (0.92–1.29)
	90–96	**1.63 (1.10–2.43)**	**1.57 (1.05–2.34)**	1.08 (0.69–1.70)	1.06 (0.67–1.66)
					
Household income	<20,000	1.12 (0.82–1.54)	0.95 (0.68–1.33)	1.28 (0.99–1.64)	1.29 (0.98–1.70)
	20,000–<50,000	**1.23 (1.05–1.43)**	1.16 (0.97–1.38)	1.15 (0.96–1.38)	1.22 (1.00–1.49)
	50,000–<100,000	1.11 (0.97–1.27)	1.13 (0.98–1.30)	1.02 (0.86–1.21)	1.08 (0.90–1.29)
	100,000–<150,000	1.07 (0.92–1.24)	1.10 (0.95–1.28)	0.99 (0.81–1.21)	1.03 (0.84–1.26)
	≥150,000 (ref.)	1.00	1.00	1.00	1.00
	Missing	1.21 (0.97–1.52)	1.12 (0.89–1.43)	1.28 (1.00–1.65)	**1.34 (1.02–1.74)**
					
Highest education attained	Secondary school diploma	1.18 (0.98–1.42)	1.15 (0.95–1.38)	1.03 (0.84–1.27)	1.03 (0.83–1.26)
	Some postsecondary	1.06 (0.96–1.18)	1.02 (0.92–1.14)	0.97 (0.86–1.09)	0.95 (0.85–1.07)
	Post-secondary diploma (ref.)	1.00	1.00	1.00	1.00
	Missing	0.96 (0.83–1.10)	0.92 (0.80–1.07)	0.94 (0.81–1.09)	0.93 (0.80–1.09)
					
Value of savings and investments	< 50,000	**1.36 (1.13–1.62)**	1.17 (0.97–1.40)	**1.41 (1.13–1.75)**	**1.30 (1.04–1.63)**
	50,000–<100,000	**1.29 (1.08–1.56)**	1.14 (0.95–1.38)	**1.32 (1.05–1.67)**	1.24 (0.98–1.57)
	100,000–<1 million	**1.18 (1.01–1.37)**	1.10 (0.94–1.28)	1.16 (0.94–1.43)	1.12 (0.91–1.39)
	≥1 million (ref.)	1.00	1.00	1.00	1.00
	Missing	**1.34 (1.08–1.67)**	**1.19 (0.95–1.49)**	**1.50 (1.15–1.95)**	**1.43 (1.09–1.87)**
					
Dwelling ownership	Renting	**1.29 (1.11–1.49)**	1.15 (0.98–1.34)	**1.21 (1.06–1.39)**	1.16 (1.00–1.34)
	Own–with mortgage	**1.15 (1.04–1.28)**	1.09 (0.97–1.22)	**1.28 (1.13–1.43)**	**1.22 (1.08–1.38)**
	Own–mortgage paid off (ref.)	1.00	1.00	1.00	1.00
					
Immigrant status	Born in Canada (ref.)	1.00	1.00	1.00	1.00
	Not born in Canada	1.05 (0.93–1.18)	1.09 (0.97–1.24)	1.12 (0.97–1.29)	1.14 (0.99–1.32)
					
Marital Status	Married/Common-law (ref.)	1.00	1.00	1.00	1.00
	Single	**1.51 (1.28–1.78)**	**1.43 (1.22–1.68)**	1.11 (0.95–1.31)	1.08 (0.92–1.27)
	Separated/Divorced/Widowed	**1.26 (1.12–1.41)**	**1.14 (1.01–1.29)**	**1.17 (1.04–1.31)**	**1.14 (1.01–1.28)**
					
Perceived community standing	1-5	**1.33 (1.18–1.50)**	**1.30 (1.16–1.47)**	**1.79 (1.57–2.05)**	**1.76 (1.54–2.01)**
	6-7	**1.22 (1.09–1.37)**	**1.22 (1.09–1.36)**	**1.32 (1.16–1.52)**	**1.31 (1.14–1.50)**
	8-10 (ref.)	1.00	1.00	1.00	1.00
					
Perception of income satisfying basic needs	Adequate (ref.)	1.00	1.00	1.00	1.00
	Inadequate	**1.69 (1.37–2.08)**	**1.58 (1.27–1.95)**	**1.89 (1.62–2.21)**	**1.83 (1.57–2.15)**
					
BMI	Underweight/normal weight (ref.)	1.00	1.00	1.00	1.00
	Overweight	0.89 (0.80–0.99)	0.98 (0.88–1.09)	0.96 (0.85–1.09)	1.01 (0.89–1.15)
	Obese	1.11 (0.98–1.24)	**1.17 (1.04–1.32)**	**1.22 (1.07–1.39)**	**1.26 (1.11–1.44)**
					
Chronic Pain	No (ref.)	1.00	1.00	1.00	1.00
	Yes	**1.51 (1.37–1.66)**	**1.48 (1.34–1.63)**	**1.57 (1.42–1.74)**	**1.56 (1.41–1.73)**
					
Multimorbidity	No chronic conditions (ref.)	1.00	1.00	1.00	1.00
	One chronic condition	**1.22 (1.10–1.36)**	**1.23 (1.11–1.36)**	1.08 (0.96–1.22)	1.10 (0.98–1.24)
	Two or more chronic conditions	**1.56 (1.37–1.77)**	**1.58 (1.39–1.80)**	**1.49 (1.31–1.69)**	**1.51 (1.32–1.72)**
					
Childhood adversities	Less than three (ref.)	1.00	1.00	1.00	1.00
	Three or more	**1.43 (1.30–1.57)**	**1.44 (1.31–1.58)**	**1.51 (1.36–1.68)**	**1.52 (1.36–1.69)**
	Missing	**1.26 (1.02–1.55)**	**1.25 (1.01–1.54)**	**1.70 (1.39–2.08)**	**1.69 (1.38–2.06)**
					
Household pet	No (ref.)	1.00	1.00	1.00	1.00
	Yes	**1.22 (1.11–1.34)**	**1.20 (1.09–1.32)**	**1.24 (1.12–1.37)**	**1.20 (1.08–1.34)**

Data are presented as OR (95% CI). Bolded ORs and 95% Cis are statistically significant. ORs for categories with less than 2% of respondents were not reported. ^a^ Adjusted for age, sex, and marital status.

**Table 4:** Adjusted odds ^a^ (95% confidence interval) of depression during Autumn 2020 among those with no history of depression, any history of depression, recent depression (during follow-up 1 wave), and distant depression (prior to follow-up 1 wave).

COVID-Related Factor		Total (*n* = 22,622)	No History of Depression (*n* = 16,525)	History of Depression (*n* = 6097)	Distant Depression (*n* = 3213)	Recent Depression (*n* = 2884)
Living alone	No (ref.)	16,716	1.00	1.00	1.00	1.00
	Yes	5527	**1.31 (1.11–1.54)**	**1.19 (1.01–1.41)**	**1.29 (1.00–1.67)**	0.98 (0.78–1.22)
						
Essential worker	No (ref.)	2396	1.00	1.00	1.00	1.00
	Yes	3279	0.69 (0.57–0.83)	0.90 (0.72–1.12)	1.10 (0.81–1.50)	0.63 (0.45–0.89)
	Missing	16,947	0.81 (0.70–0.93)	1.01 (0.85–1.20)	1.03 (0.81–1.31)	0.84 (0.64–1.11)
						
COVID health stressors	No (ref.)	7295	1.00	1.00	1.00	1.00
	Yes	15,222	**1.64 (1.48–1.81)**	**1.37 (1.22–1.53)**	**1.35 (1.15–1.60)**	**1.28 (1.09–1.52)**
						
COVID resource access	No (ref.)	3660	1.00	1.00	1.00	1.00
	Yes	18,857	**1.68 (1.48–1.91)**	**1.67 (1.45–1.92)**	**1.59 (1.30–1.95)**	**1.75 (1.41–2.17)**
						
COVID family conflict	No (ref.)	1992	1.00	1.00	1.00	1.00
	Yes	20,525	**5.13 (4.45–5.92)**	**3.57 (3.01–4.23)**	**3.89 (3.06–4.94)**	**3.06 (2.38–3.94)**
						
COVID other family issues	No (ref.)	12,811	1.00	1.00	1.00	1.00
	Yes	9706	**1.72 (1.55–1.91)**	**1.57 (1.39–1.76)**	**1.53 (1.29–1.81)**	**1.63 (1.38–1.93)**
						
COVID health care	No (ref.)	5514	1.00	1.00	1.00	1.00
	Yes	17,003	**1.90 (1.70–2.11)**	**1.77 (1.56–2.00)**	**1.81 (1.52–2.16)**	**1.66 (1.39–1.99)**
						
Lack companionship	No (ref.)	14,191	1.00	1.00	1.00	1.00
	Yes	8377	**5.00 (4.49–5.55)**	**4.19 (3.70–4.74)**	**4.15 (3.48–4.95)**	**3.66 (3.05–4.38)**
						
Feeling left out	No (ref.)	16,119	1.00	1.00	1.00	1.00
	Yes	6345	**5.66 (5.10–6.28)**	**4.92 (4.36–5.55)**	**4.99 (4.20–5.94)**	**4.26 (3.57–5.07)**
						
Feeling isolated	No (ref.)	12,960	1.00	1.00	1.00	1.00
	Yes	9584	**5.32 (4.78–5.94)**	**4.49 (3.97–5.08)**	**4.27 (3.58–5.10)**	**4.31 (3.59–5.16)**

^a^ Odds ratios are adjusted for age, sex, value of savings, marital status, chronic pain, and experiences of childhood adversities.

**Table 5:** Crude and adjusted odds ratios and 95% confidence intervals of being depressed during Autumn 2020 based on pre-pandemic history of depression status and sex.

By Pre-Pandemic History of Depression Status	Depressed at Autumn 2020				Crude OR (95% CI)	Adjusted OR (95% CI)
	No	%	Yes	%		
No history of depression	14,397	87.1	2128	12.9	1.00	1.00
Any history of depression	3342	54.8	2755	45.2	5.58 (5.21–5.97)	3.79 (3.48–4.12) ^a^
Distant history of depression	2136	66.5	1077	33.5	3.41 (3.13–3.72)	2.57 (2.31–2.85) ^a^
Recent history of depression	1206	41.8	1678	58.2	9.41 (8.63–10.27)	5.87 (5.27–6.55) ^a^
**By sex**						
Male						
No history of depression	7501	89.5	881	10.5	1.00	1.00
Any history of depression	1308	59.4	893	40.6	5.81 (5.21–6.49)	4.11 (3.59–4.71) ^b^
Distant history of depression	849	71.3	342	28.7	3.43 (2.97–3.96)	2.68 (2.25–3.18) ^b^
Recent history of depression	459	45.4	551	54.6	10.22 (8.87–11.78)	6.62 (5.55–7.90) ^b^
Female						
No history of depression	6896	84.7	1247	15.3	1.00	1.00
Any history of depression	2034	52.2	3109	47.8	5.06 (4.64–5.52)	3.60 (3.23–4.01) ^b^
Distant history of depression	1287	63.6	735	36.4	3.16 (2.83–3.52)	2.49 (2.18–2.85) ^b^
Recent history of depression	747	39.9	1127	60.1	8.34 (7.47–9.32)	5.46 (4.76–6.27) ^b^

^a^ Adjusted model includes age, sex, value of savings, marital status, chronic pain, experience of childhood adversity, loneliness (feeling left out), living alone during COVID-19, and COVD-19 related stressors. ^b^ Adjusted model includes age, value of savings, marital status, chronic pain, experience of childhood adversity, loneliness (feeling left out), living alone during COVID-19, and COVD-19 related stressors.

## Data Availability

Data are available from the Canadian Longitudinal Study on Aging (www.clsa-elcv.ca) for researchers who meet the criteria for access to de-identified CLSA data.
